# Asking and Receiving

**Published:** 1888-03-24

**Authors:** H. Merivale


### ASKING AND RECEIVING.

A shelter—spent and tempest-driven
Mid winter's strife,—
I sought, and found the boon of heaven,
Eternal life.
Oh Word, how is thy truth confessed!
Who seeketh part shall find the whole;
I asked but for the wanderer's rest,
And found the traveller's goal.

I asked some kindly door to ope for
My weary head;
The heart of Love I dared not hope for
Stood wide instead.
Oh Word, how is thy truth confessed!
Who sues for little, all has won;
I, that would be thy winter-guest,
Was thy belovéd son.

—*H. Merivale.*

